# Development in Danish medical students’ empathy: study protocol of a cross-sectional and longitudinal mixed-methods study

**DOI:** 10.1186/s12909-020-1967-2

**Published:** 2020-02-19

**Authors:** E. Assing Hvidt, J. Søndergaard, N. C. Hvidt, S. Wehberg, A. Büssing, C. M. Andersen

**Affiliations:** 1 Research Unit of General Practice, Department of Public Health, University of Southern Denmark, J.B. Winsløwsvej 9 A, 5000 Odense, Denmark; 2 Department for the Study of Culture, University of Southern Denmark, Campusvej 55, 5230 Odense M, Denmark; 3 Institute of Integrative Medicine, Witten/Herdecke University, Gerhard-Kienle-Weg 4, 58313 Herdecke, Germany; 4 Department for Psychology, University of Southern Denmark, Campusvej 55, 5230 Odense M, Denmark

**Keywords:** Study protocol, Empathy, Compassion, Altruism, Medical education, Medical students, Cross-sectional study, Longitudinal study, Qualitative study, Focus group interviews

## Abstract

**Background:**

Clinical empathy has been associated with positive outcomes for both physicians and patients such as: more accurate diagnosis and treatment, increased patient satisfaction and compliance, and lower levels of burnout and stress among physicians. International studies show mixed results regarding the development of empathy among future physicians associating medical education with decline, stability or increase in empathy levels. These mixed results are due to several study limitations. In Denmark, no investigation of Danish medical students’ empathy trajectory has yet been conducted wherefore such a study is needed that optimizes the study design of earlier studies.

**Methods:**

The aim of the study is to examine and analyze empathy levels and empathy changes among Danish medical students from the four medical faculties in Denmark, employing a cross-sectional and longitudinal mixed-methods design including a control group of non-medical students. By supplementing cross-sectional and longitudinal questionnaire studies with a focus group interview study it is the aim to identify and analyze factors (including educational) that are perceived by medical students to influence the development of empathy and its expression in clinical care.

**Discussion:**

The results of the study will provide insight into the trajectory of medical students’ empathy and in undergraduate and graduate students’ experiences with and perceptions of empathy development. In addition, the study will provide evidence to support further research on how targeted educational programmes can best be designed to educate empathic and patient-centered physicians.

## Background

In this study we aim to identify and analyse empathy scores and empathy developments among Danish medical students. Empathy is recognized as a multidimensional construct involving a cognitive, emotional and behavioral component [1, 2]. Cognitive empathy involves an intellectual understanding of another’s state of mind without any personal emotional response [1]. Emotional empathy on the other hand is commonly viewed as an emotional resonance with another’s emotional state, e.g. through compassion as an intention to help. The behavioral component refers to concrete actions in response to the aforementioned intention to relieve the person’s distress, commonly referred to as prosocial or even altruistic behavior [3, 4].

Defined in the medical literature as the ability to understand a patient’s suffering and concerns combined with an ability to communicate this understanding and an intention to help [5–7], clinical empathy has been associated with a number of beneficial patient and physician outcomes: more accurate diagnosis and treatment, increased patient satisfaction and compliance [6, 8–11], lower incidence of complaints and lawsuits, and lower levels of burnout and stress among physicians [5, 12, 13]. Moreover, high scores on empathy among medical students are associated with the following positive outcomes: increased satisfaction with their education, lower levels of stress and burn-out, higher ratings of overall clinical competences given by medical school faculty, better interpersonal skills assessed by patients and greater teamwork skills [5, 14–16]. However, as future physicians, they are confronted with increasing work stress and contact with suffering patients which may result in burnout indicators such as emotional exhaustion and emotional withdrawal. For that reason, future physicians must find strategies to protect their own functionality [17]. One of the major tasks in medical education is to maintain or increase clinical empathy among medical students and to help them find and train strategies to cope with these future stressors that might also decrease their motivation in the clinical routine.

Yet, according to several research studies that measure empathy in the context of medical education, a significant decline in empathy occurs among medical students as their training progresses. These studies have been conducted in disparate cultural settings, e.g. including the UK [18], USA [19–22], Iran [23, 24], Kuwait [25], India [26], the Caribbean [27] and China [28]. The decrease in empathy among medical students documented in some studies have created concern among educational managers in medical faculties asking themselves what has gone wrong [29]. Several factors have been suggested to contribute to a development towards increased emotional distance and detachment among medical students in their contact to patients: increased time pressures, increased patient encounters and an increased awareness of evidence-based medicine protocols and technologies, the influence of senior role models and the hidden curriculum [29]. In order to enhance empathy as an ability, compassion as an intention and altruism as concrete behaviour, biopsychosocial, patient-centered and relationship-centered illness models have been integrated into the medical curricula in a number of countries alongside the biomedical paradigm [30, 31]. Moreover, students engage in various learning initiatives that have empathy cultivation and preservation as a key goal, such as accompanying patients on medical visits, making home visits, and reading medically related literature and poetry (narrative medicine) [32–35].

However, other studies, e.g. in Portugal [36], the UK [37], Kuwait [38], Japan [39], Korea [40], Bangladesh [41] and New Zealand [42], find either no difference or an increase in empathy, hereby challenging the narrative about medical education and empathy decline. No study about Danish medical students’ empathy levels has yet been conducted.

The mixed and ambiguous results of the above-mentioned wide-ranging international studies are possibly related to study limitations and cultural and contextual differences between cohorts. Although existing studies have contributed with significant knowledge about the trajectory of empathy in medical education, they call for research that optimizes the measurement of empathy, addressing/identifying also factors that determine and influence the development of empathy [12].

So far, most international studies have relied upon single, self-report instruments, mostly on the widely used measure Jefferson Scale of Physician Empathy-Student version (JSE-S), developed specifically to measure empathy in respect to patient care, reflecting primarily the cognitive dimension of empathy. This neglects the fact that empathy is constituted by different components and dimensions [12]. Furthermore, many studies have been single-institutional and cross-sectional without use of controls making it difficult to state with certainty that the changes measured are generalizable, progressive, systematic and specific to medical students. Existing studies also lack consideration on determinant factors related to, e.g. personal experience, personality structure, stress perception, coping abilities, values and psychological well-being that may influence levels of empathy.

To avoid some of these pitfalls, we employ triangulation in this study, using more than one instrument to measure empathy and related constructs such as compassion and altruism, and using both quantitative and qualitative methods. Thus, we combine a quantitative cross-sectional and longitudinal research design with a cross-sectional qualitative design in order to uncover more deeply why and how changes in empathy might occur in and across the four Danish universities. Furthermore, we use a large control group of non-medical students, enabling understanding of the level of empathy that medical students have in relation to their age-related non-medical university student peers. Furthermore, empathy measurement among students in the control group enables us to investigate whether empathy level is predictive of choice of study and dropout.

## Methods/design

### Aim and research questions

The aim of the study is to examine and analyze empathy scores and empathy developments among Danish medical students from four different universities based on a quantitative self-assessment questionnaire and qualitative focus group discussions.

The specific research questions are:

1. Which are the differences in empathy scores between 1st, 3rd and 6th year medical students in Denmark and as compared to non-medical students from one of the four universities?
2. Which factors do medical students perceive to influence the development in empathy and its expression in clinical care?
3. What are the developments in medical students’ self-assessed empathy over the course of their training?
4. What are the associations between students’ levels of empathy, compassion, altruism and stress, attachment patterns, personal values, conscience presence and self-control (situational mindfulness) and lastly, religiosity?

The study is a mixed methods study incorporating the following three interrelated sub-studies (studies 1–3) each of them relating to the above-mentioned research questions:

*Study 1* is designed as a national, cross-sectional study including 1st, 3rd and last year medical students from all four universities in Denmark (University of Copenhagen, Aarhus University, University of Southern Denmark and Aalborg University). A control group of 1st, 3rd and 6th year students from the University of Southern Denmark will be invited to participate.

*Study 2* is designed as a qualitative focus group study. The focus group method has been chosen for data generation because its interactional features might promote a dynamic exchange of the students’ opinions and experiences [43, 44]. Specifically, we wish to involve the students in a reflection about 1) understandings and expressions of empathy, 2) their own experiences with the role of empathy in patient care and 3) contextual, including educational, factors that might impact on their empathy.

*Study 3* is designed as a national, longitudinal study where students from the 1st year of study 1 will be followed over time in order to examine empathy scores over time. The students will receive the questionnaire described under study 1 each year during their medical education.

### Pilot study

In order to evaluate the technical, administrative or logistic feasibility of the study, including issues of questionnaire design and data collection, we conducted a pilot study in June 2019 at the University of Southern Denmark. Students in their 1st, 3rd and 6th year were invited to participate in the project via an invitation letter in e-Boks - an online digital mailbox that is linked to the personal registration number of each Danish citizen. Descriptive analyses of the pilot study are presented in the results section.

### Study settings and samples

All 1st, 3rd and 6th year medical students from Denmark’s four universities are invited to participate constituting sample one, including approximately 4.300 students. All 1st, 3rd and 6th year non-medical students enrolled at University of Southern Denmark are invited to participate in the control group constituting sample two, including approximately 10.000 students.

### Data collection

Quantitative data is collected 1st of March 2020 through an online questionnaire. Students are informed about the study by receiving an information letter, which clarifies how their data is used according to the General Data Protection Regulation (GDPR) and gives them the contact information on the principle investigators. Furthermore, the contact information of the data protection officers of the faculty is provided. Students are informed that their participation in the study is voluntary and that, if they do not want to participate, dropout analysis will be conducted. The questionnaire will be set up in SurveyXact (electronic survey system). Complying with European data protection rules, the University of Southern Denmark approved the data processing activities regarding this project, including permission to extract the students’ Central Person Register number and other relevant background information such as year, study start, grades, country origin, dropout, etc. from the faculties’ enrolment lists, and registered the project under [Journal no. 10.181].

In the questionnaire, the students will be asked to fill in demographic and background information about their gender, age, number of children, and relationship status. Purpose-designed questions have been developed to examine the students’ own experiences with the healthcare system and their medical education.

The following nine scales are included in the questionnaire in the listed order:

1. *The Jefferson Scale of Empathy* – Student Version (JSE-S) [22]. A 20-item scale developed to specifically measure medical students’ personal orientation toward empathy in respect to patient care. The students’ response is measured on a 7-point Likert scale. The scale has been extensively validated internationally and has shown a stable factor structure [23, 28, 38, 39, 45–47].
2. *The Interpersonal Reactivity Index* (IRI) [48] consists of 28 items measuring four aspects of empathy in the general population: perspective taking, fantasy, empathic concern and personal distress. The students’ response is measured on a 5-point Likert scale. In several validation studies undertaken in different settings, the IRI has been found a valid instrument showing a stable factor structure [49].
3. *The Santa Clara Brief Compassion Scale* (SCBCS) [50] is a 5-item scale rated on a 7-point Likert scale. The scale measures compassion and its relation to pro-social behaviours and has demonstrated good reliability and validity [51].
4. *The Generative Altruism Scale* (GAIS) [52], measures both affective and behavioural elements of altruism. Altruism is defined here as an attitude and commitment to help and care for others without expecting any rewards or direct benefit, with altruism being intrinsically motivated by compassion. The scale consists of 11 items rated on a 4-point rating scale and has demonstrated good reliability and validity [52].
5. Attachment is measured with *the Experiences in Close Relationship* (ECR)–Short Form [53] which is a 12-item questionnaire rated on a 7-point Likert scale. The scale includes two subscales measuring attachment anxiety and attachment avoidance. The reliability, test-retest and construct validity of the scale have been found acceptable [53]. Attachment will be measured at all time points in study 1, and in the 3rd and 6th year in study 3.
6. *The Valued Living Questionnaire (VLQ)* [54] is an instrument that examines 10 valued domains of living: 1. Family, 2. Marriage/couples/intimate relations, 3. Parenting, 4. Friendship, 5. Work, 6. Education, 7. Recreation, 8. Spirituality, 9. Citizenship, and 10. Physical self-care. The 10 areas of life are rated on a scale of 1–10, indicating the level of importance and how consistently the respondents have lived in accord with those values in the past week.
7. *The Perceived Stress Scale* (PSS) [55] measures the degree to which situations in one’s life are appraised as stressful. PSS is a 10-item questionnaire rated on a 4-point Likert scale and the most widely used psychological instrument for measuring the perception of stress. The PSS has been validated in a Danish context [56]. The PSS is added to measure if perceived stress affect students’ levels of empathy.
8. *Conscious Presence and Self Control (CPSC)* [57] is a 10-item validated scale that measures a person’s situational awareness (‘mindfulness’) in both daily life and difficult situations, and subsequent reaction of being consciously ‘present’ and non-judgemental. Items are rated on a Likert scale with the following response options: “rarely” (0), “occasionally” (1), “fairly often” (2), and “almost always” (3). The scale has a good internal consistency.
9. *The Centrality of Religiosity Scale (CRS)* [58] available in different versions (with 5, 7, 10 and 15 items) measures the centrality, importance or salience of religious meanings in personality. It measures five core dimensions of religiosity: public practice, private practice, ideological, intellectual and religious experience. Validation studies have shown adequate overall psychometric qualities [59]. In this study, the 7-item version is used.

The PSS and VLQ already exist in a Danish-language translation [56].^1^ The remaining seven scales have been translated into Danish according to the WHO’s guidelines [60] and cognitive interviews have been conducted with ten Danish medical students prior to the pilot study [61].

The control group of non-medical students will receive a closely related copy of the questionnaire where only the JSE-S and purpose-designed questions specific to the medical education are removed. Permission has been obtained for using the following scales: JSE-S, SCBCS, GAIS, VLQ, CPSC and CRS. The remaining scales are freely accessible online with no copyright stated: IRI, ECR and PSS.

Qualitative data are generated through focus group interviews with medical students from the four universities. The interactional features of the focus group method [44] will facilitate the students’ collective engagement with understandings of and experiences with empathy in the students’ personal lives, during their education and in the clinical encounters. The focus group interviews are guided by a topic guide containing open-ended questions that relate to the research question, such as what empathy could mean and how the students themselves understand it, experiences with its expression in clinical care, perceived empathy development during their education, perceived educational factors influencing the level and practice of empathy, etc. We seek to include approximately 72 students from study 1 (1st, 3rd and 6th year students) into the study who are distributed in 12 focus groups with six students in each group (one group per year per university). The students are recruited through advertisements in student Facebook forums, on student portals and during classes. The students who show interest in participating are then selected purposefully based on the desire to achieve diversity in gender, age, and geographical place of residence. The focus group discussions will last approximately 60–75 min and are digitally recorded and transcribed verbatim hereafter.

### Outcomes

Primary outcomes are self-reported empathy scores as measured with scale one and two (JSE-S and IRI) within a cross-sectional and longitudinal comparison and knowledge about student-perceived beneficial and impeding factors for empathy as generated through focus group interviews. Secondary outcomes are compassion and altruism as the intentional and behavioural outcomes of empathy, stress perception as a putative challenge of students’ empathy, attachment, personal values, conscience presence and self-control as a suggested resource to buffer stress and protect their empathy and intentions to help, and religiosity as a suggested resource which may motivate empathy, compassion and altruism. These outcomes are measured by the above-mentioned scales three to nine (SCBCS, GAIS, ECR, VLQ, PSS, CPSC, CRS).

### Data analysis

The primary analysis for the quantitative data from study 1 and 3 will be based on separate linear regression models for the two primary outcome measures JSE-S and IRI. In study 1, we will focus on the effect of time and student group (medical versus non-medical) on the outcomes, but also take into account the effect of other covariates such as gender, age, study year, grades, etc. The longitudinal nature of study 3 implies the possibility to model individual change in empathy levels over time, while adjusting for covariates as stated above. Also, when appropriate, we will calculate the effect size estimates (e.g., Cohen d) in order to examine whether statistically significant differences in empathy scores are practically (clinically) significant [62]. Throughout the analyses, a *p*-value below 0.05 will be considered statistically significant.

The qualitative data from focus group interviews will be transcribed and analysed using a thematic content analysis approach [63, 64] and by means of the software program NVivo 12. The transcripts will be coded in two phases: an initial open coding and a subsequent closed thematic coding focusing on socially constructed understandings and how these affect behaviours [65] using a node structure reflecting identified themes and subthemes allowing for expansion and reduction along the way. Those of the researchers who have coded the data will discuss and agree upon the identified themes at analytic meetings (intercoder agreement) [66], relating them to the original transcripts and aligning them where necessary.

### Results from the pilot study

A total of 862 medical students received an invitation to participate in the pilot study of which 258 completed the questionnaire completely (30%), 47 only partially and 557 did not respond. Those who completed the questionnaire were more often female, under the age of 25 years and 1st year students (Table 1). Table 2 depicts the students’ score on the included scales, showing the range of scores, mean, SD, min, 25th percentile, median 75th percentile and maximum.

**Table 1:** Participants’ characteristics of the pilot study

Variables	Total (*N* = 862)	Completed (*N* = 258)	Partly completed (*N* = 47)	Non-response (*N* = 557)
Male N(%)	324 (100.0)	75 (23.1)	17 (5.2)	232 (71.6)
Female N(%)	538 (100.0)	183 (34.0)	30 (5.6)	325 (60.4)
Age: 18–25 N(%)	497 (100.0)	157 (31.6)	31 (6.2)	309 (62.2)
Age: > = 25 N(%)	365 (100.0)	101 (27.7)	16 (4.4)	248 (67.9)
^a^Quota 1 N(%)	427 (100.0)	124 (29.0)	24 (5.6)	279 (65.3)
^a^Quota 2 N(%)	395 (100.0)	126 (31.9)	21 (5.3)	248 (62.8)
^a^Quota missing N(%)	40 (100.0)	8 (20.0)	2 (5.0)	30 (75.0)
1st year N(%)	326 (100.0)	113 (34.7)	17 (5.2)	196 (60.1)
3rd year N(%)	277 (100.0)	81 (29.2)	16 (5.8)	180 (65.0)
6th year N(%)	259 (100.0)	64 (24.7)	14 (5.4)	181 (69.9)

^a^Quota refers to how the students are enrolled into the universities. Quota 1 is based exclusively on the students’ marks whereas Quota 2 takes into account other qualifying aspects as related work or volunteer experiences

**Table 2:** Descriptive data of the scale response of the students who completed the whole questionnaire

Scales	N	Range	Mean	SD	Min	25th percentile	Median	75th percentile	Max
Empathy									
JSE-S	258	20–140	91.3	7.6	61.0	87.0	92.0	97.0	106.0
IRI_PT	258	0–28	19.8	4.0	8.0	18.0	20.0	23.0	28.0
IRI_F	258	0–28	18.8	5.2	4.0	15.0	19.0	23.0	28.0
IRI_EC	258	0–28	20.3	4.7	5.0	17.0	21.0	24.0	28.0
IRI_PD	258	0–28	7.9	4.3	0.0	5.0	8.0	11.0	22.0
Compassion									
SCBCS	258	5–35	25.2	5.9	6.0	22.0	26.0	29.0	35.0
Altruism									
GAIS	258	0–3	1.4	0.4	0.1	1.1	1.5	1.7	2.9
Attachment^a^									
ECR-S	216	12–84	32.9	9.6	13.0	26.0	31.0	40.0	63.0
ECR-S_Anxiety	216	6–42	21.0	6.6	7.0	16.5	20.0	25.0	42.0
ECR-S_Avoidance	216	6–42	11.9	5.6	6.0	7.0	11.0	14.5	41.0
Values									
VLQ	258	20–200	147.2	20.0	83.0	133.0	148.0	162.0	191.0
Perceived stress									
PSS	258	0–40	15.4	6.6	0.0	11.0	14.0	20.0	36.0
Conscious Presence and Self Control									
CSPC	258	0–3	1.7	0.5	0.5	1.4	1.7	2.0	2.9
Religion									
CRSi-7	258	1–5	2.0	0.7	1.0	1.4	1.9	2.4	4.9

^a^Only students who had or had had a partner answered the ECR (42 answered no to having og having had a partner)

*IRI* Interpersonal Reactivity Index, Subscales of the IRI: *IRI_PT* perspective taking, *IRI_F* fantasy, *IRI_EC* empathic concern, *RIR_PD* personal distress, *JSE-S* Jefferson Scale of Physician Empathy student version, *SCBCS* The Santa Clara Brief Compassion Scale, *GAIS* the generative altruism scale, *ECR-S* experiences in close relationship – short form, *VLQ* the valued living questionnaire, *PSS* perceived stress scale, *CSPC* conscious presence and self control, *CRSi*-7 centrality of religiosity scale – 7 items

## Discussion

Results generated will provide us with knowledge about 1. Differences in empathy scores between 1st, 3rd and 6th year Danish medical students and between medical and non-medical students, 2. significant educational and cultural factors influencing the development of empathy as perceived by Danish medical students, 3. the progressive potential change in empathy levels in Danish medical students from their 1st to 6th year and 4. associations between empathy and the following variables: compassion, altruism, stress, attachment, personal values, conscience presence and self-control and religiosity.

These results will be an important contribution to research in empathy in medical education and will provide the medical faculties with evidence to support further research on how targeted educational programs can be designed to retain, cultivate and enhance empathy among medical students and students of other health professions.

Knowledge from the above-mentioned pilot study will also be incorporated in the main study. For example, the response rate of the pilot study was low (30%). Partly, this was due to the timing of the study which was in June where some of the students had already gone on summer vacation and others were busy preparing for exams. Based on this knowledge, the questionnaire for study 1 will be sent out 1st of March 2020 when the students have just begun the semester. Further strategies to increase the response rate include: 1) showing a short film at key introductory lectures at the four universities explaining the aim of the study, 2) adding small professional drawings to the questionnaire to uphold the students’ motivation and 3) sending out three reminders.

## Data Availability

The datasets generated and/or analyzed during the current study are not publicly available.

## Abbreviations

CPSC: Conscious Presence and Self Control
CRS: The Centrality of Religiosity Scale
ECR: Experiences in Close Relationship
GAIS: The Generative Altruism Scale
IRI: The Interpersonal Reactivity Index
JSE-S: The Jefferson Scale of Empathy – Student Version
PSS: The Perceived Stress Scale
SCBCS: The Santa Clara Brief Compassion Scale
VLQ: The Valued Living Questionnaire

## Footnotes

1 The VLQ has been translated by psychologist Rikke Kjeldgaard.

## References

[1] Hojat M (2016). Empathy in health professions education and patient care: springer.

[2] Spreng RN, McKinnon MC, Mar RA, Levine B (2009). The Toronto empathy questionnaire: scale development and initial validation of a factor-analytic solution to multiple empathy measures. J Pers Assess.

[3] Kunyk D, Olson JK (2001). Clarification of conceptualizations of empathy. J Adv Nurs.

[4] Schantz ML (2007). Compassion: a concept analysis. Nurs Forum.

[5] Hojat M, Vergare M, Isenberg G, Cohen M, Spandorfer J (2015). Underlying construct of empathy, optimism, and burnout in medical students. Int J Med Educ.

[6] Derksen F, Bensing J, Lagro-Janssen A (2013). Effectiveness of empathy in general practice: a systematic review. Br J Gen Pract.

[7] Mercer SW, Reynolds WJ (2002). Empathy and quality of care. Br J Gen Pract.

[8] Epstein RM, Hadee T, Carroll J, Meldrum SC, Lardner J, Shields CG (2007). “could this be something serious?” reassurance, uncertainty, and empathy in response to patients’ expressions of worry. J Gen Intern Med.

[9] Hellstrom O (1998). Dialogue medicine: a health-liberating attitude in general practice. Patient Educ Couns.

[10] Neumann M, Edelhäuser F, Kreps G, Scheffer C, Lutz G, Tauschel D (2010). Can patient–provider interaction increase the effectiveness of medical treatment or even substitute it? An exploration on why and how to study the specific effect of the provider. Patient Educ Couns.

[11] Undeland M, Malterud K (2008). Diagnostic interaction: the patient as a source of knowledge?. Scand J Prim Health Care.

[12] Quince T, Thiemann P, Benson J, Hyde S (2016). Undergraduate medical students’ empathy: current perspectives. Adv Med Educ Pract.

[13] Derksen F, Bensing J, Kuiper S, van Meerendonk M, Lagro-Janssen A (2015). Empathy: what does it mean for GPs? A qualitative study. Fam Pract.

[14] Hojat M, Gonnella JS, Mangione S, Nasca TJ, Veloski JJ, Erdmann JB (2002). Empathy in medical students as related to academic performance, clinical competence and gender. Med Educ.

[15] Hojat M, Bianco JA, Mann D, Massello D, Calabrese LH. Overlap between empathy, teamwork and integrative approach to patient care. Med Teach. 2014:1–4. 10.3109/0142159X.2014.971722 25314019

[16] Berg K, Majdan JF, Berg D, Veloski J, Hojat M (2011). A comparison of medical students’ self-reported empathy with simulated patients’ assessments of the students’ empathy. Med Teach.

[17] Büssing A, Falkenberg Z, Schoppe C, Recchia DR, Poier D (2017). Work stress associated cool down reactions among nurses and hospital physicians and their relation to burnout symptoms. BMC Health Serv Res.

[18] Austin EJ, Evans P, Magnus B, O'Hanlon K (2007). A preliminary study of empathy, emotional intelligence and examination performance in MBChB students. Med Educ.

[19] Hojat M, Mangione S, Nasca TJ, Rattner S, Erdmann JB, Gonnella JS (2004). An empirical study of decline in empathy in medical school. Med Educ.

[20] Chen D, Lew R, Hershman W, Orlander J (2007). A cross-sectional measurement of medical student empathy. J Gen Intern Med.

[21] Hojat M, Vergare MJ, Maxwell K, Brainard G, Herrine SK, Isenberg GA (2009). The devil is in the third year: a longitudinal study of erosion of empathy in medical school. Acad Med.

[22] Hojat M, Gonnella JS (2015). Eleven years of data on the Jefferson scale of empathy-medical student version (JSE-S): proxy norm data and tentative cutoff scores. Med Princ Pract.

[23] Shariat SV, Habibi M (2013). Empathy in Iranian medical students: measurement model of the Jefferson scale of empathy. Med Teach..

[24] Khademalhosseini M, Khademalhosseini Z, Mahmoodian F (2014). Comparison of empathy score among medical students in both basic and clinical levels. J Adv Med Educ Prof.

[25] Hasan S, Al-Sharqawi N, Dashti F, AbdulAziz M, Abdullah A, Shukkur M (2013). Level of empathy among medical students in Kuwait University, Kuwait. Med Princ Pract.

[26] Shashikumar R, Chaudhary R, Ryali VS, Bhat PS, Srivastava K, Prakash J (2014). Cross sectional assessment of empathy among undergraduates from a medical college. Med J Armed Forces India.

[27] Youssef FF, Nunes P, Sa B, Williams S (2014). An exploration of changes in cognitive and emotional empathy among medical students in the Caribbean. Int J Med Educ.

[28] Wen D, Ma X, Li H, Liu Z, Xian B, Liu Y (2013). Empathy in Chinese medical students: psychometric characteristics and differences by gender and year of medical education. BMC Med Educ..

[29] Shapiro J (2008). Walking a mile in their patients’ shoes: empathy and othering in medical students’ education. Philos Ethics Humanit Med.

[30] Borrell-Carrió F, Suchman AL, Epstein RM (2004). The biopsychosocial model 25 years later: principles, practice, and scientific inquiry. Ann Fam Med.

[31] Frankel RM (2004). Relationship-centered care and the patient-physician relationship. J Gen Intern Med.

[32] Dow AW, Leong D, Anderson A, Wenzel RP (2007). Using theater to teach clinical empathy: a pilot study. J Gen Intern Med.

[33] Charon R (2000). Reading, writing, and doctoring: literature and medicine. Am J Med Sci.

[34] Kelm Z, Womer J, Walter JK, Feudtner C (2014). Interventions to cultivate physician empathy: a systematic review. BMC Med Educ..

[35] Batt-Rawden SA, Chisolm MS, Anton B, Flickinger TE (2013). Teaching empathy to medical students: an updated, systematic review. Acad Med.

[36] Magalhães E, Salgueira AP, Costa P, Costa MJ (2011). Empathy in senior year and first year medical students: A cross-sectional study. BMC Med Educ.

[37] Tavakol S, Dennick R, Tavakol M (2011). Empathy in UK medical students: differences by gender, medical year and specialty interest. Educ Prim Care.

[38] Roh M-S, Hahm B-J, Lee DH, Suh DH (2010). Evaluation of empathy among Korean medical students: a cross-sectional study using the Korean version of the Jefferson scale of physician empathy. Teach Learn Med.

[39] Kataoka HU, Koide N, Ochi K, Hojat M, Gonnella JS (2009). Measurement of empathy among Japanese medical students: psychometrics and score differences by gender and level of medical education. Acad Med.

[40] Lee BK, Bahn GH, Lee WH, Park JH, Yoon TY, Baek SB (2009). The relationship between empathy and medical education system, grades, and personality in medical college students and medical school students. Korean J Med Educ.

[41] Mostafa A, Hoque R, Mostafa M, Rana MM, Mostafa F (2014). Empathy in undergraduate medical students of Bangladesh: psychometric analysis and differences by gender, academic year, and specialty preferences. ISRN Psychiatry.

[42] Quince TA, Kinnersley P, Hales J, da Silva A, Moriarty H, Thiemann P (2016). Empathy among undergraduate medical students: a multi-Centre cross-sectional comparison of students beginning and approaching the end of their course. BMC Med Educ.

[43] Morgan DL, Holstein JA, Gubrium JF (2001). Focus group interviewing. Handbook of interview research: Context and method.

[44] Liamputtong P (2011). Focus group methodology: principle and practice: sage publications limited.

[45] Preusche I, Wagner-Menghin M (2013). Rising to the challenge: cross-cultural adaptation and psychometric evaluation of the adapted German version of the Jefferson scale of physician empathy for students (JSPE-S). Adv Health Sci Educ.

[46] Stansfield RB, Schwartz A, O'Brien CL, Dekhtyar M, Dunham L, Quirk M (2016). Development of a metacognitive effort construct of empathy during clinical training: a longitudinal study of the factor structure of the Jefferson scale of empathy. Adv Health Sci Educ Theory Pract.

[47] Ferreira-Valente A, Costa P, Elorduy M, Virumbrales M, Costa MJ, Pales J (2016). Psychometric properties of the Spanish version of the Jefferson scale of empathy: making sense of the total score through a second order confirmatory factor analysis. BMC Med Educ..

[48] Davis MH (1983). Measuring individual differences in empathy: evidence for a multidimensional approach. J Pers Soc Psychol.

[49] Manarte L (2017). Towards a new structure of the interpersonal reactivity index. Reliability and validation of the Portuguese version: a comparative analysis. Eur Psychiatry.

[50] Hwang JY, Plante TG, Lackey K (2008). The development of the Santa Clara brief compassion scale: an abbreviation of Sprecher and Fehr’s compassionate love scale. Pastoral Psychol.

[51] Plante TG, Mejia J (2016). Psychometric properties of the Santa Clara brief compassion scale. Pastoral Psychol..

[52] Büssing A, Kerksieck P, Günther A, Baumann K (2013). Altruism in adolescents and young adults: validation of an instrument to measure generative altruism with structural equation modeling. Int J Childrens Spiritual.

[53] Wei M, Russell DW, Mallinckrodt B, Vogel DL (2007). The experiences in close relationship scale (ECR)-short form: reliability, validity, and factor structure. J Pers Assess.

[54] Wilson KG, Murrell AR, Hayes SC, Follette VM, Linehan MM (2004). Values work in acceptance and commitment therapy: Setting a course for behavioral treatment. Mindfulness and acceptance: Expanding the cognitive behavioral tradition.

[55] Cohen S, Kamarck T, Mermelstein R (1983). A global measure of perceived stress. J Health Soc Behav.

[56] Eskildsen A, Dalgaard VL, Nielsen KJ, Andersen JH, Zachariae R, Olsen LR (2015). Cross-cultural adaptation and validation of the Danish consensus version of the 10-item perceived stress scale. Scand J Work Environ Health.

[57] Büssing A, Walach H, Kohls N, Zimmermann F, Trousselard M (2013). Conscious Presence and Self Control as a measure of situational awareness in soldiers - A validation study. Int J Ment Heal Syst.

[58] Huber S, Huber OW (2012). The centrality of religiosity scale (CRS). Religions..

[59] Esperandio M, August H, Viacava J, Huber S, Fernandes M (2019). Brazilian Validation of Centrality of Religiosity Scale (CRS-10BR and CRS-5BR). Religions.

[60] World Health Organisation. Process of translation and adaptation of instruments. 2018 [22 May 2018]. Available from: http://www.who.int/substance_abuse/research_tools/translation/en/.

[61] Garcia AA (2011). Cognitive interviews to test and refine questionnaires. Public Health Nurs.

[62] Hojat M, Xu G (2004). A visitor’s guide to effect size: Statistical versus practical (clinical) importance of research findings. Adv Health Sci Educ Theory Pract.

[63] Denzin NK, Lincoln YS (1994). The handbook of qualitative research.

[64] Silverman D (2001). Interpreting qualitative data. Methods for analyzing talk, text and interaction.

[65] Berger P, Luckmann T (1966). The social construction of identity.

[66] Creswell JW. Qualitative inquiry and research design: choosing among five approaches: Sage; 2013.

[67] World Medical Association Declaration of Helsinki (2013). Ethical principles for medical research involving human subjects. JAMA..

[68] National Videnskabsetisk Komité [National Committee on Health Research Ethics]. Hvad skal jeg anmelde? [What to notify?] 2020 [Available from: http://en.nvk.dk/how-to-notify/what-to-notify.

